# Supplementary material for: Larval Dispersal Modeling Reveals Low Connectivity among National Marine Protected Areas in the Yellow and East China Seas
Source: Biology (Basel). 2023 Mar 2;12(3):396. doi: 10.3390/biology12030396 (PMC10044727; doi:10.3390/biology12030396)
Supplement: Supplementary file 1 [file biology-12-00396-s001.zip › biology-2122151-supplementary.pdf]

# Supplementary Materials for

## Larval dispersal modeling reveals low connectivity among national marine protected areas in the Yellow and East China Seas

**This file includes:**

- Table S1. Source of biological parameters for study species
- Figure S1-S4. Potential larval export of species with different pelagic larval duration
- Figure S5. Self-recruitment fraction and subsidy recruitment fraction of MPAs
- Figure S6. The computational grid of the ROMS model and the domain of configuration area
- Particle stability analysis for recent eight years

**Table S1. Source of biological parameters for study species**

| Scientific name | PLD （days） | Depth （m） | Spawning window | Source |
|---|---|---|---|---|
| *Nemipterus virgatus* | 75 | 25 | Apr-May | https://www.fishbase.se/summary/Nemipterus-virgatus.html |
| *Sepiella maindroni* | 45 | 10 | Apr-May | WU C, DONG Z, CHI C, et al. (2010) [1] |
| *Epinephelus bruneus* | 45 | 20 | May-Jun | WANG B, SUN P, ZHANG Z, et al. (2006) [2] |
| *Larimichthys crocea* | 33 | 15 | Apr-May | ZHUANG P. (2006) [3] |
| *Argyrosomus argentatus* | 33 | 50 | Jun-Aug | https://www.fishbase.de/Summary/SpeciesSummary.php?ID=434&AT=%26%2330333%3B%26%2322993%3B%26%2340060%3B |
| *Argyrosomus japonicus* | 33 | 100 | Jan-Mar | WANG B, ZHANG X, QU X, et al. (2002) [4] |
| *Evynnis cardinalis* | 30 | 45 | Nov-Jan | CAI Y, CHEN Z, XU Z, et al. (2017) [5] |
| *Apostichopus japonicus* | 20 | 10 | May-Jun | https://www.reeflex.net/tiere/6815_Apostichopus_japonicus.htm |
| *Scomberomorus niphonius* | 19 | 20 | May-Jun | Tang Q. (2012) [6] |
| *Portunus Trituberculatus* | 17 | 20 | Apr-Jun | CHUL O. (2011) [7] |
| *Epinephelus akaara* | 15 | 25 | Apr-Jun | M UKAWA. (1966) [8] |
| *Anguilla japonica* | 10 | 3 | Nov-Dec | ZOU Y. (2002) [9] |
| *Penaeus japonicus* | 10 | 20 | Dec-Mar | CAI X. (1981) [10] |
| *Acropora solitaryensis* | 7 | 5 | Apr-May | ZHANG Y, HUANG H, HAUNG J, et al. (2013) [11] |

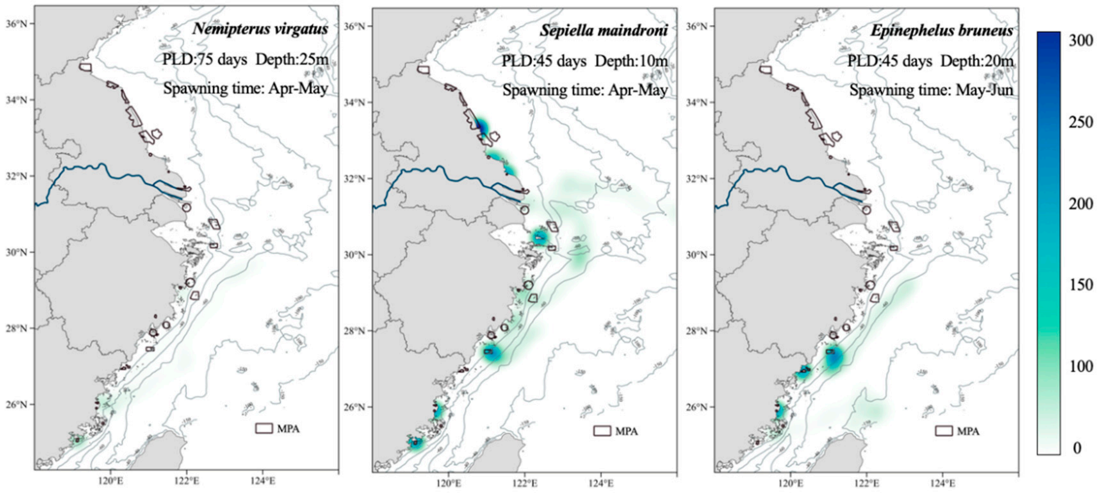


**Figure S1**. Potential larval export of species with PLD⩾45: *Nemipterus virgatus, Sepiella maindroni, Epinephelus bruneus*

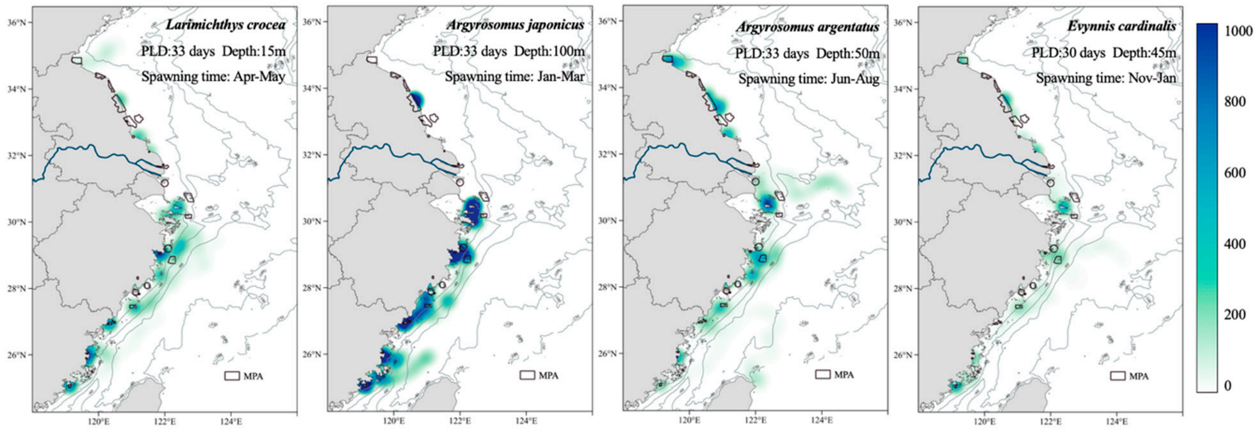


**Figure S2.** Potential larval export of species with PLD (30~44): *Larimichthys crocea, Argyrosomus argentatus, Argyrosomus japonicus, Evynnis cardinalis*

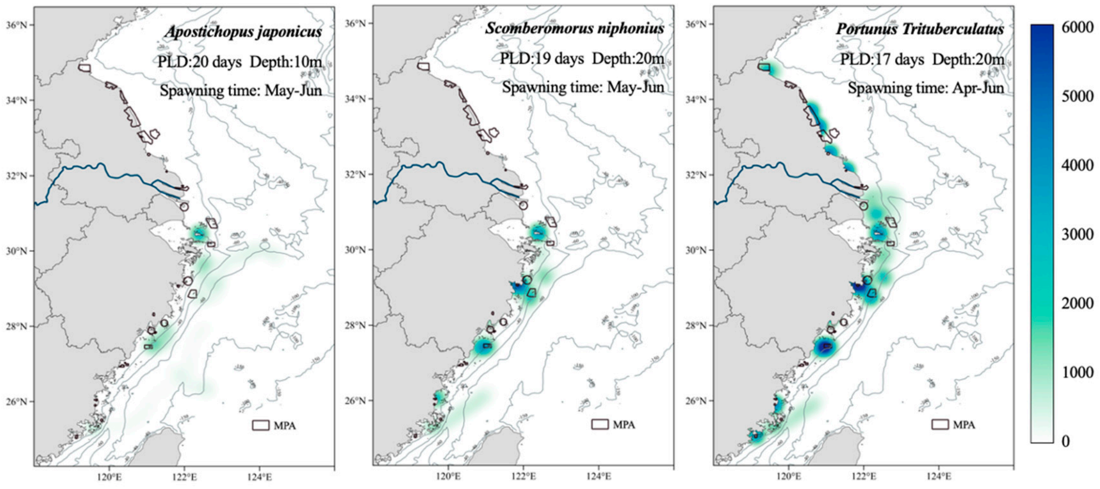


**Figure S3.** Potential larval export of species with PLD (16~29): *Apostichopus japonicus, Scomberomorus niphonius, Portunus Trituberculatus*

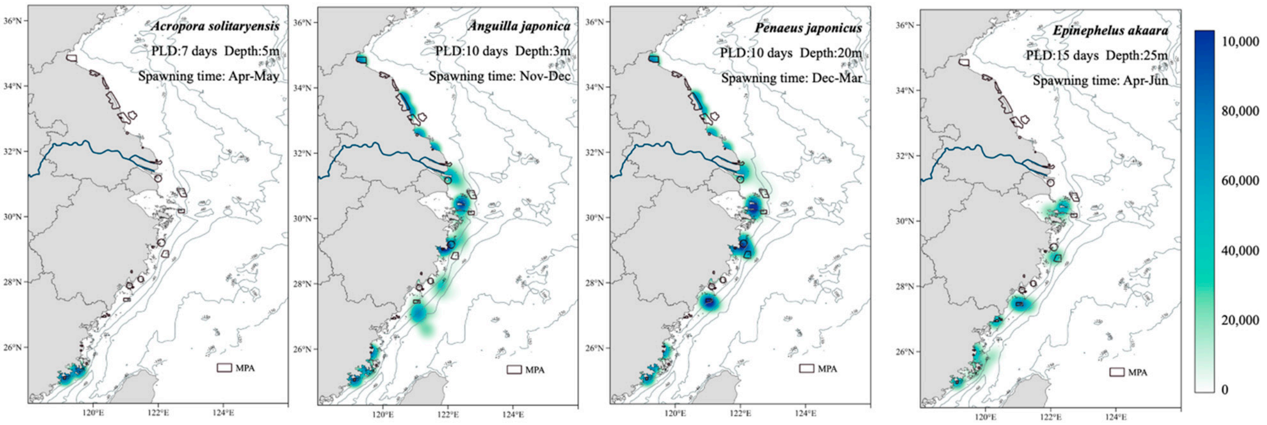


**Figure S4.** Potential larval export of species with short PLD (⩽15): *Epinephelus akaara, Anguilla japonica, Penaeus japonicus, Acropora solitaryensis*

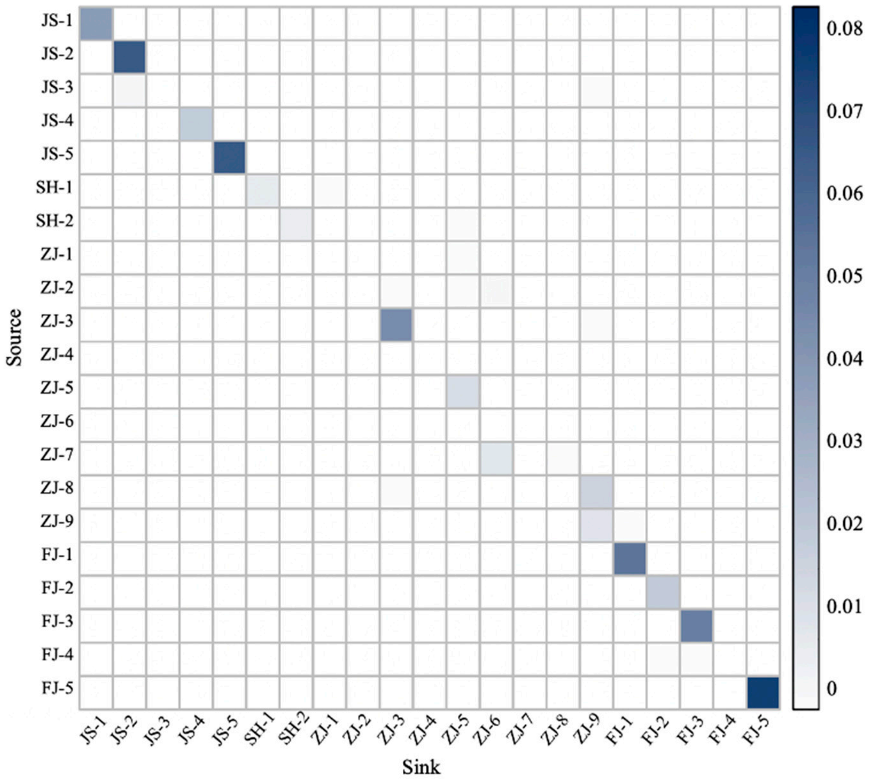


**Figure S5.** Self-recruitment fraction and subsidy recruitment fraction of MPAs

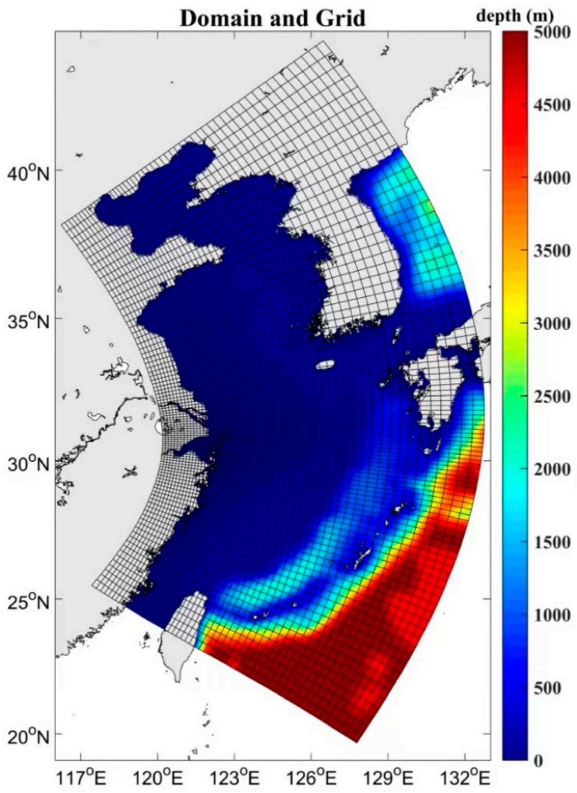


**Figure S6.** The computational grid of the ROMS model and the domain of configuration area.

## Particle stability analysis for recent eight years

The reason for selecting the year 2016 because the particle dispersal dynamics in 2016 basically was an average field while there can be differences from year to year.

We have verified the rationality of selecting the year 2016 through simple model test when we built the model in 2019. We did Lagrangian particle tracking with altimeter-derived geostrophic velocities using 4th order Runge-Kutta method with Dormand-Prince pairs. We selected Shengsi SMPA (ZJ-1) as the release point and July 1st as the release start time. The particles floated for seven days. The simulation was conducted based on eight years of surface geostrophic satellite data from 2014 to 2018(https://data.marine.copernicus.eu/products?option=com_csw&task=results). In order to see the particle stability of each year, we divided every three years into a group and mapped the positions of three points from different groups at the same time to a plane. Then we calculated the area of the circumcircle of the triangle formed by them, so as to see the degree of dispersion of the three points.

The results (the figure below) showed that the particle dispersion of all the other groups showed relatively stable except for the group 2014-2016. The reason why the three particles from 2014-2016 were really discrete could be attributed to the strong El Niño which caused dramatic changes in the global climate. Besides, the group 2015-2017 was more stable than the group 2016-2018. Since the model was

built in 2019, the data of 2016 was relatively new at the time. Based on the above results, we considered that the particle dispersal was more stable in 2016 during 2014-2018.

In conducting this study, we updated the particle stability test by 2021. The particles from group 2017-2019 and the group 2018-2020 were the most stable while the particles from group 2019-2021 were more discrete than group 2015-2017. Compared with the group 2014-2016 and 2016-2018, the circumcircle areas of other groups were relatively close. The results indicated that besides the year 2016, the particle dispersal dynamics of the years after 2018 could also be considered average fields in recent eight years.

Of course, the selection still has some limitations. First, we admit that the model test was simple, considering only one release point and short floating time. The results could only show the changes in the coastal current near the release point and the preliminary judgment on the particle stability of different years. Second, several researches and our study simulated for one year [12,13] while some new studies have begun to explore long-term simulations and predict impacts of future climate change on MPA connectivity[14-16]. There could be heterogeneous connectivity patterns during the spawning events at inter-annual scales[15]. Climate change can lead to reduced pelagic larval duration and larvae survival rate, affecting population connectivity and the optimal location of MPAs[16]. If larval dispersal simulation can be carried out in coastal China for years, these studies will provide more reliable references for Marine protection in China to cope with future climate change.

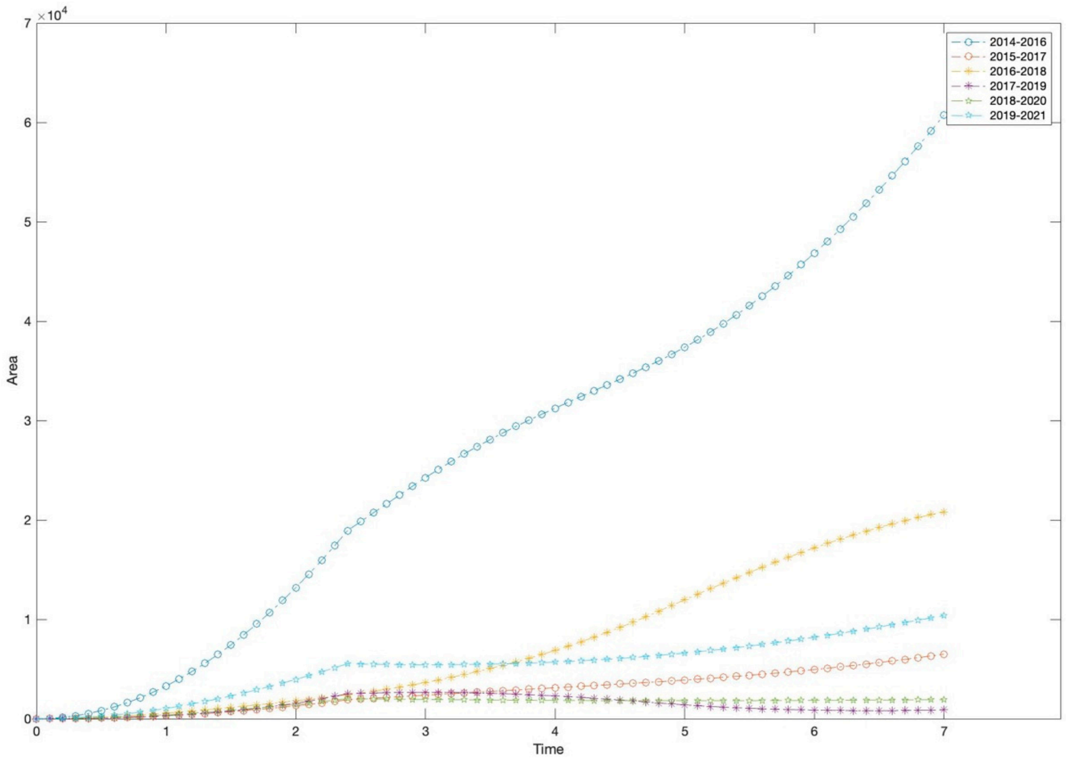


**Figure S7.** The circumcircle areas of the triangles formed by particles for recent eight years (2014-2021)

## References


1. Wu, C.; Dong, Z.; Chi, C.; Ding, F.Reproductive and spawning habits of *Sepiella maindroni* off Zhejiang, China. *Oceanologia et Limnologia Sinica.* **2010**, *41*, 39-46.

2. Wang, B.; Sun, P.; Zhang, Z.; Ma, J. Biological characteristics of *Epinephelus brunneus* and preliminary experiment of indoor culture. *Fishery Modernization.* **2006**, 1, 28-29.

3. Zhuang, P. *Yangtze Estuary fishes*; Shanghai Science and Technology Press: China, 2006; Volume 10, pp. 338-340.

4. Wang, B.; Zhang, X.; Qu, X.; Ruan, Sh.; Qu, Y. Biological characteristics and technologies of seed production of Japanese croaker (N. Japanica). *Progress in Fishery Sciences.* **2002**, 4, 13-19.

5. Cai, Y.; Chen, Z.; Xu, Sh.; Zhang, K. Tempo-spatial distribution of Evynnis cardinalis in Beibu Gulf. *South China Fisheries Science*. **2017**, *13*, 1-10.

6. Tang, Q. *Regional oceanography of China Seas-Fisheries oceanography*; China Ocean Press: China, 2012; Volume 6, pp. 166-169.

7. Oh, C.-W. Population Biology of the Swimming Crab Portunus Trituberculatus (Miers, 1876) (Decapoda, Brachyura) on the Western Coast of Korea, Yellow Sea. *Crustaceana* **2011**, *84*, 1251-1267.

8. Ukawa, M.; Higuchi, M.; Mito, S. Spawning habits and early life history of a serranid fish, Epinephelus akaara (TEMMINCK et SCHLEGEL). *Japanese Journal of Ichthyology* **1966**, *13*, 156-161.

9. Zou, Y. *Special aquaculture* ; China Agriculture Press: China, 2002; Volume 07, pp. 30-31.

10. Cai, X.; Lin, Q.; Wan, W. On the Development of the Prawn *Penaeus penicillatus* in Comparison with Both *P.japonicus* and *P. orientalis*. *Journal of Xiamen University(Natural Science).* **1981**,2, 243-252.

11. Zhang, Y.; Huang, H.; Huang, J.; Yuan, T. Experimental cultivation of coral larvae in Xisha Islands. *Ocean Development and Management.* **2013**, *30*, 78-82.

12. Liu, G.; Bracco, A.; Quattrini, A.; Herrera, S. Kilometer-Scale Larval Dispersal Processes Predict Metapopulation Connectivity Pathways for Paramuricea biscaya in the Northern Gulf of Mexico. *Frontiers in Marine Science* **2021**, *8*, 790927.

13. Andrello, M.; Mouillot, D.; Beuvier, J.; Albouy, C.; Thuiller, W.; Manel, S. Low Connectivity between Mediterranean Marine Protected Areas: A Biophysical Modeling Approach for the Dusky Grouper Epinephelus marginatus. *Plos One* **2013**, *8*, e68564.

14. Assis, J.; Fragkopoulou, E.; Serrão, E.A.; Horta e Costa, B.; Gandra, M.; Abecasis, D. Weak biodiversity connectivity in the European network of no-take marine protected areas. *Science of The Total Environment* **2021**, *773*, 145664.

15. Lopera, L.; Cardona, Y.; Zapata-Ramírez, P.A. Circulation in the Seaflower Reserve and Its Potential Impact on Biological Connectivity. *Frontiers in Marine Science* **2020**, *7*, doi:10.3389/fmars.2020.00385.

16. Rassweiler, A.; Ojea, E.; Costello, C. Strategically designed marine reserve networks are robust to climate change driven shifts in population connectivity. *Environmental Research Letters* **2020**, *15*, 034030.
